# Role of Large Cabbage White butterfly male-derived compounds in elicitation of direct and indirect egg-killing defenses in the black mustard

**DOI:** 10.3389/fpls.2015.00794

**Published:** 2015-09-29

**Authors:** Nina E. Fatouros, Luis R. Paniagua Voirol, Fryni Drizou, Quyen T. Doan, Ana Pineda, Enric Frago, Joop J. A. van Loon

**Affiliations:** ^1^Laboratory of Entomology, Wageningen UniversityWageningen, Netherlands; ^2^Institute of Biology, Dahlem Centre of Plant Sciences, Freie Universität BerlinBerlin, Germany; ^3^Division of Plant and Crop Sciences, School of Biosciences, Sutton Bonington Campus, The University of NottinghamNottingham, UK

**Keywords:** hypersensitive response, induced plant defenses, oviposition-induced plant volatiles, egg parasitoid, Brassicaceae, *Pieris*accessory reproductive glands, *PR-1*

## Abstract

To successfully exert defenses against herbivores and pathogens plants need to recognize reliable cues produced by their attackers. Up to now, few elicitors associated with herbivorous insects have been identified. We have previously shown that accessory reproductive gland secretions associated with eggs of Cabbage White butterflies (*Pieris* spp.) induce chemical changes in Brussels sprouts plants recruiting egg-killing parasitoids. Only secretions of mated female butterflies contain minute amounts of male-derived anti-aphrodisiac compounds that elicit this indirect plant defense. Here, we used the black mustard (*Brassica nigra*) to investigate how eggs of the Large Cabbage White butterfly (*Pieris brassicae*) induce, either an egg-killing direct [i.e., hypersensitive response (HR)-like necrosis] or indirect defense (i.e., oviposition-induced plant volatiles attracting *Trichogramma* egg parasitoids). Plants induced by *P. brassicae* egg-associated secretions expressed both traits and previous mating enhanced elicitation. Treatment with the anti-aphrodisiac compound of *P. brassicae*, benzyl cyanide (BC), induced stronger HR when compared to controls. Expression of the salicylic (SA) pathway- and HR-marker *PATHOGENESIS-RELATED GENE1* was induced only in plants showing an HR-like necrosis. *Trichogramma* wasps were attracted to volatiles induced by secretion of mated *P. brassicae* females but application of BC did not elicit the parasitoid-attracting volatiles. We conclude that egg-associated secretions of *Pieris* butterflies contain specific elicitors of the different plant defense traits against eggs in *Brassica* plants. While in Brussels sprouts plants anti-aphrodisiac compounds in *Pieris* egg-associated secretions were clearly shown to elicit indirect defense, the wild relative *B. nigra*, recognizes different herbivore cues that mediate the defensive responses. These results add another level of specificity to the mechanisms by which plants recognize their attackers.

## Introduction

Plants form the base of most terrestrial ecosystems and are under attack by a diverse community of insect herbivores. Natural selection has shaped interactions between these two trophic levels such that plants have evolved anti-herbivore defenses, and herbivores have evolved strategies to better exploit their hosts. To understand the evolution of plant – insect interactions it is crucial to know how insect attack is perceived and in particular which signals are involved in this dialog (Bonaventure et al., 2011). Upon feeding damage, plant cells produce damage-associated molecular patters (DAMPs) which trigger a general plant wound response (Heil, 2009). Recognition of more specific effectors in these interactions is based on so-called herbivore-associated molecular patterns (HAMPs), which may be present in, e.g., oral or oviposition secretions (Felton and Tumlinson, 2008; Mithöfer and Boland, 2008; Acevedo et al., 2015; Hilker and Fatouros, 2015). Several effectors like amino acid-lipid conjugates, peptides, or enzymes originating from herbivore salivary glands have been shown to elicit direct plant defenses that can reduce herbivore performance or indirect defenses that improve the foraging of natural enemies (Schoonhoven et al., 2005; Bonaventure et al., 2011; Felton et al., 2014). The plant hormones jasmonic acid (JA), ethylene (ET), and salicylic acid (SA) have been shown to be the main players in orchestrating the downstream production of defense compounds effective against chewing insects (Wu and Baldwin, 2010; Erb et al., 2012; Thaler et al., 2012). Yet, relatively little is known on the recognition of HAMPs by plants compared to interactions between plants and microbes, oomycetes, or nematodes, for which hundreds of elicitors have been described (Felton et al., 2014).

Many plant-insect interactions do not start with a feeding herbivore, but with an ovipositing female. As a cue that signals future damage, egg recognition and triggering of the appropriate response might provide plants with fitness benefits (Pashalidou et al., 2015). Several plant species have been shown to respond to herbivore egg deposition by expressing direct or indirect egg-killing defenses (Hilker and Fatouros, 2015). The ability of recognizing this early phase of attack might be crucial for plant survival but its consequences have been often neglected when studying the ecology of associations between herbivorous insects and plants (Hilker and Fatouros, 2015). A few effectors of oviposition-induced plant defenses have been identified and isolated from egg-associated secretions and extracts from homogenized eggs (Reymond, 2013; Hilker and Fatouros, 2015). Insects have also evolved strategies to overcome plant recognition; there is evidence that eggs might contain effector molecules that suppress plant defenses (Bruessow et al., 2010). More insights into plant responses to these egg-derived elicitors is required to understand their ecological consequences and potential evolutionary importance.

In brassicaceous plants, different oviposition-induced direct and indirect defenses have been identified. Brussels sprouts plants (*Brassica oleracea* var. *gemmifera*) induced by egg deposition of two Cabbage White butterfly species, the Large Cabbage White *Pieris brassicae* and the Small Cabbage White *P. rapae* were shown to retain *Trichogramma* egg parasitoids by chemical modifications in the leaf surface (Fatouros et al., 2005, 2009; Pashalidou et al., 2010), a type of indirect plant defense using so-called substrate-borne chemical cues (Colazza et al., 2014). During mating, pierid males transfer species-specific anti-aphrodisiac compounds in their spermatophores to females. These compounds were shown to curtail courtship and decrease the likelihood of female re-mating (Andersson et al., 2000, 2003). In previous work by Fatouros et al. (2008, 2009), we showed that egg-associated secretions produced by accessory reproductive glands (ARGs) of mated females of Cabbage White butterflies contain minute amounts of anti-aphrodisiac compounds that induced the biosynthesis of chemical cues retaining *Trichogramma* wasps. A subsequent study in *Arabidopsis thaliana* showed that epicuticular waxes, in particular some fatty acids, might act as cues for the wasps (Blenn et al., 2012).

**FIGURE 1:**
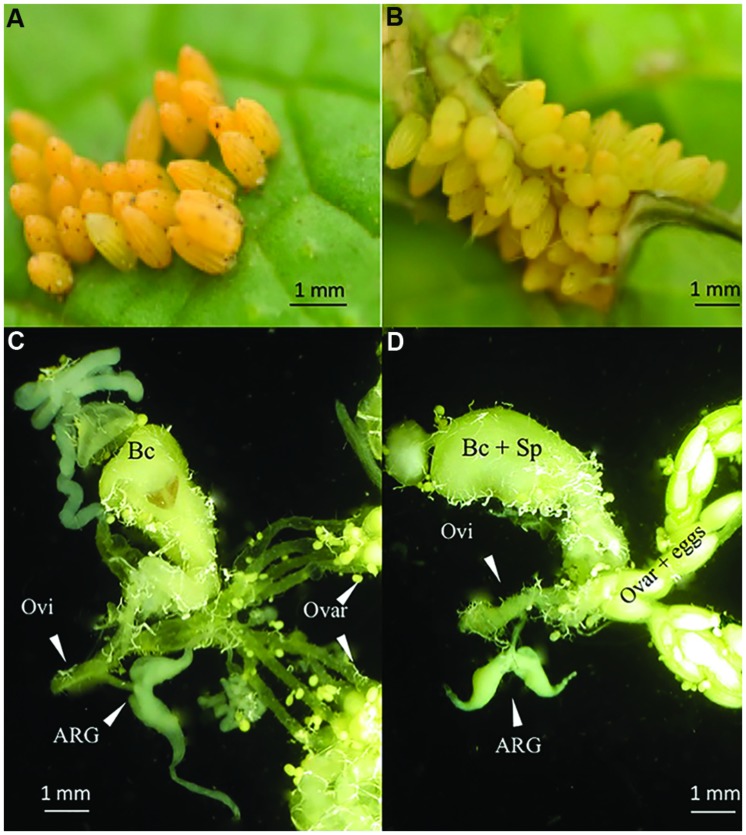
**Hypersensitive response (HR)-like necrosis of *Brassica nigra* plants induced by *Pieris* eggs and female reproductive tract. (A)** *Pieris brassicae* egg clutch on a leaf showing no necrosis (HR-; credits: E. Griese), **(B)** *P. brassicae* egg clutch inducing strong necrosis (HR+; credits: E. Griese), **(C)** reproductive tract of a virgin *P. brassicae* female, and **(D)** reproductive tract of a mated *P. brassicae* female. Ovi, oviduct; ARG, accessory reproductive gland; Ovar, ovariole; Bc, bursa copulatrix; Sp, spermatheca.

*Brassica nigra* is an annual plant that responds to *P. brassicae* eggs by a necrosis resembling pathogen-induced hypersensitive response (HR) (**Figures 1A,B**), and which can directly kill eggs by desiccating or dropping-off the plant (Shapiro and De Vay, 1987; Fatouros et al., 2012, 2014). Furthermore, *B. nigra* plants were shown to release volatile chemical cues induced by eggs of the gregarious *P. brassicae* that attract egg and larval parasitoids and repel conspecific butterflies (Fatouros et al., 2012; Cusumano et al., 2015; Ponzio et al., manuscript accepted). Interestingly, in the closely related but solitary *P. rapae*, both HR-like necrosis and oviposition-induced plant volatiles (OIPVs) were shown to act synergistically, leading to high egg mortality in nature (Fatouros et al., 2014). However, the elicitors responsible for these two traits in *B. nigra* are still unknown.

The aim of this study is to investigate whether egg-killing defenses in *B. nigra* are induced by ARG secretions released by the ovipositing female and whether anti-aphrodisiac compounds transferred during mating play a role in the elicitation. We conducted experiments to investigate whether (i) the elicitor of HR and OIPVs induced by *P. brassicae* is located in the ARGs, (ii) mating plays a role in elicitation, and if so (iii) whether the male-derived anti-aphrodisiac compound benzyl cyanide (BC) triggers HR and/or the emission of OIPVs attracting egg parasitoids. For a better understanding of the mechanistic basis of these interactions, we further tested whether (iv) JA and SA signaling pathways were involved in plant responses to *P. brassicae* egg deposition and the anti-aphrodisiac compound BC by measuring their activity using two marker genes: *lipoxygenase gene2* (*LOX2*) and *pathogenesis-related gene1* (*PR1*).

## Materials and Methods

### Plants and Insects

Seeds of black mustard plants (*B. nigra* L.) were either obtained from a population collected along the river Rhine in Wageningen, The Netherlands or provided by the Centre for Genetic Resources [CNG, (accession number: CGN06619), Wageningen, The Netherlands]. The latter were multiplied by exposing them to pollinators in the surroundings of Wageningen. Both plant populations have been used in previous studies on *Pieris* oviposition-induced defenses (Fatouros et al., 2012, 2014). The plants were grown in a greenhouse (18 ± 5°C, 50–70% RH, L16:D8), and used in the experiments when 3–5 weeks old. The Large Cabbage White butterfly *P. brassicae* L. (Lepidoptera: Pieridae) was reared on Brussels sprouts plants (*B. oleracea* var. *gemmifera* cv. Cyrus) at 21 ± 1°C, 50–70% RH, and photoperiod L16:D8, and mated females obtained from a cage where up to 50 adults of similar age were allowed to mate. Females that were observed mating were selected and were used in the experiments either immediately or 2 days after, depending on the experiment. Virgin females were obtained by isolating females immediately after eclosion (Fatouros et al., 2009). The isofemale line ED16 of *Trichogramma evanescens* Westwood (Hymenoptera: Trichogrammatidae) was reared on *Ephestia kuehniella* eggs (Koppert, Berkel en Rodenrijs, The Netherlands) under standardized conditions in a climate chamber (25 ± 1°C, 50–70% r.h., L16:D8). Newly emerged male and female wasps were kept together for 2–5 days to allow mating and then used in the preference experiments. All wasps used in the preference experiments lacked previous contact with any plant material or host residues and are referred to as inexperienced.

### Preparation of ARG Homogenates

Accessory reproductive glands (ARGs) were dissected from mated and virgin *P. brassicae* females (3–6 days after eclosion) (**Figures 1C,D**) under a stereo-microscope (optical magnification 20×) in phosphate buffered saline (PBS). One gland was transferred to 100 μL PBS, homogenized and centrifuged and used to treat a single plant.

### Plant Treatments

**FIGURE 2:**
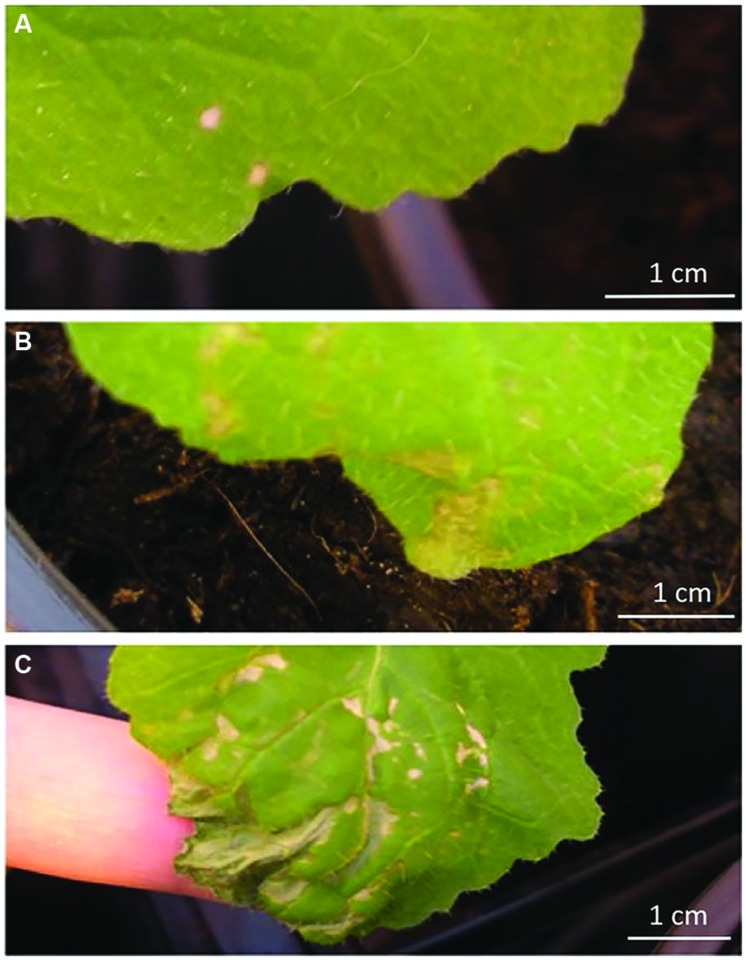
**Different severities of HR-like necrosis after application of ARG homogenate or anti-aphrodisiac compounds**. Surface area of necrotic tissue and intensity of necrosis was assessed using a relative scale: **(A)** weak (HR+): no or little necrotic tissue on upper leaf side, **(B)** medium (HR++): larger parts of treated area with brownish necrotic tissue on upper leaf side, and **(C)** strong necrosis (HR+++): almost whole treated area necrotic on both sides.

To obtain plants with 1–3 *P. brassicae* egg clutches, individual *B. nigra* plants were placed in a cage with ca. 50 *P. brassicae* males and females for up to 10 min until egg deposition was observed, and then they were transferred to the greenhouse (18 ± 5°C, 50–70% RH, L16:D8) for either four or 24 h depending on whether they were used in the bioassays or gene expression analysis, respectively. In bioassays on the effect of *P. brassicae* ARG homogenate on HR, 100 μl of the ARG supernatant of either mated or virgin females and PBS as control was applied with a fine brush to the edge of three different leaves of the same plant. For bioassays or gene expression analyses with plants treated with the anti-aphrodisiac compound BC, solutions of 0.5, 1, and 1.5 ng of BC/100 μl or the solvent ethanol (70%) as control were made. The solutions were applied with a fine brush to the edge of the third or fourth leaf from the top. For all plants, development of the necrotic tissue (HR) was checked after 72 h and visually categorized as none, weak, medium, or strong necrosis (**Figure 2**). A total of eight replicated plants were used per treatment. For bioassays on elicitation of egg parasitoid-attracting volatiles, either ARG homogenate of *P. brassicae* females (mated or virgin) or BC (1 or 10 ng/100 μl ethanol) was applied on *B. nigra* plants. Test plants were compared with control plants treated with the solvent (PBS as control for ARG or ethanol for the anti-aphrodisiac) in a Y-tube olfactometer (see below) 24 h after treatment.

### Y-tube Olfactometer Tests

We tested the attraction of *T. evanescens* wasps to volatiles of *B. nigra* plants induced by either ARG homogenates of *P. brassicae* or their anti-aphrodisiac compound (BC). Bioassays were conducted by using a dynamic airflow Y-tube olfactometer (For details see Fatouros et al., 2012). This olfactometer was designed to track minute wasps like *Trichogramma* sp. so that they can be released in groups. We have previously established that group-release does not influence the behavior of *Trichogramma* wasps (Fatouros et al., 2012). Ten adult females were released and their choice for one of the two odor sources was recorded. Thereafter the position of the odor sources was exchanged and another group of 10 wasps was released to test their preference for the same two odor sources. After 45 min, the wasps present in the collection flasks placed at the end of the arms section of the olfactometer were counted. The wasps that did not make a choice within 45 min were recorded as “no response”. The odor sources tested were plants treated with (i) ARG homogenates of either virgin or mated (2 or 48 h after mating) females against control plants treated with the solvent PBS, (ii) BC at two different concentrations (1 and 10 ng/100 μl ethanol), according to the concentrations that were detected in ARGs of mated *P. brassicae* females in a previous study (Fatouros et al., 2008), against control plants treated with ethanol only. In addition, we recorded the presence or absence of a hypersensitive-like response of the infested plants that were used in the bioassay. Per odor source combination 4–7 different plants with one replicate per experimental day were tested with 20 wasps released per replicate (80–140 wasps/ treatment). Each wasp was used only once.

### Quantitative RT-PCR Analysis of Defense Genes

We used available or cloned primers for *B. nigra* of one gene encoding lipoxygenase (*LOX2*), a key enzyme in the biosynthesis of JA in *Arabidopsis* (Bell et al., 1995), and one pathogenesis-related (PR) gene, shown for *Arabidopsis* to function downstream of the SA signaling pathway (*PR1*) (Jirage et al., 2001). The used sequences of *B. nigra* gene specific primers were: *PATHOGENESIS-RELATED* GENE1 *PR1*_F_ 5′- CTTGGCCATGGGTAGCGGCG-3′ and *PR1*_R_ 5′-ACACCTCGCTTTGCCACATCCA-3′, *LIPOXYGENASE* GENE2 *LOX2*_F_ 5′-TGCTCGTGCACGCCAGAGTC-3′ and *LOX2*_R_ 5′-AGCCAGCCCCCTGCTGATGA-3′. Total RNA was extracted from app. 100 mg liquid nitrogen-ground leaf powder using the RNeasy Plant Mini kit (Qiagen). One μg of total RNA was treated with DNaseI (Invitrogen) and subsequently transcribed into cDNA using the iScript cDNA synthesis kit (Biorad) following the manufacturer’s protocol. Quantitative RT-PCR was performed in a Rotor-Gene 6000 machine (Corbett Research) with a 72-well rotor. The amplification reactions were performed in 25 μl final volume containing 12.5 μl iQ SYBR Green Supermix (Bio-Rad, Hercules, CA, USA), 300 nM of the gene-specific forward and reverse primer, and 5 μl cDNA. All qRT-PCR experiments were performed in duplicate and average values were used in the analyses. The following PCR program was used for all PCR analyses: 95°C for 3 min, followed by 40 cycles of 95°C for 15 s, and 45 s at 62°C. Ct values were normalized by using *GAPDH* as a reference gene (primer sequences: F 5′-GCTACGCAGAAGAC AGTTGATGG-3′ and R 5′-TGGGCACACGGAAGGACATAC-3′). Normalized gene expression was then calculated as 2^-ΔΔCt^ (Livak and Schmittgen, 2001). Data from 3 to 4 biological replicates were used for statistical analysis. For each biological replicate, two leaf disks 1 cm away from the treated area were collected from each plant as described above.

### Statistics

The occurrence of HR on the plants and its intensity was analyzed with generalized linear models with a binomial error distribution corrected for overdispersion using the glm command and the quasibinomial family in R (R 3.0.2 http://www.r-project.org/). For both the effect of the females’ gland extract and the concentration of BC, two different analyses were performed: one considering the occurrence of HR as a binomial response (i.e., no HR compared with all other categories), and another as no, or weak HR compared with medium or strong HR. To test for the effect of the females’ gland extract, control, virgin, or mated females were included as categorical fixed factors, and for the effect of the BC the different concentrations were also included as categorical fixed factors. When overall differences were found, pairwise differences between factors were also tested applying the Bonferroni adjustment to the significance level. Data from the Y-tube olfactometer were analyzed by expressing the number of wasps that chose the test odor as the fraction of all responding wasps and arcsine transforming the variable. Subsequently, choices were tested against a 50:50 distribution with a one-sample *t*-test. Gene transcription data were log-transformed for normalization, and analyzed with one-way ANOVA for the two time points independently. Then the Dunnett test was used for *post hoc* comparisons between the different treatments and the control treatment.

## Results

### Location of HR Elicitor and Effect of Mating and Male-derived Compounds

**FIGURE 3:**
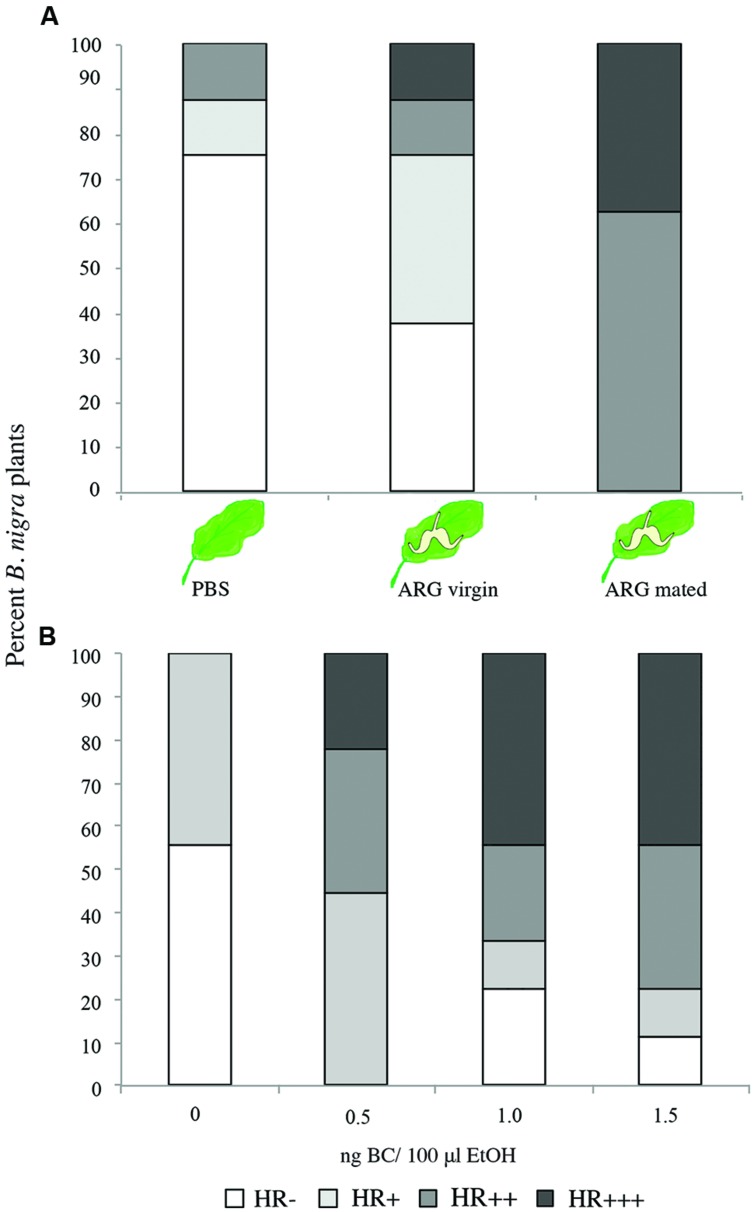
**Percentage of *B. nigra* plants treated with ARG secretion from *P. brassicae* females or anti-aphrodisiac compound expressing HR-like necrosis. (A)** treatments with ARG secretions from *P. brassicae* females (*n* = 8 plants/treatment, all treatments on one plant), **(B)** treatments with anti-aphrodisiac compound from *P. brassicae* females, benzyl cyanide (BC) at three different concentrations 0.5–1.5 ng/100 μl ethanol (EtOH) or EtOH only (*n* = 9 plants/ treatment. HR-: no necrosis observed, HR+ to +++: necrosis, which was assessed using a relative scale as shown in **Figures 1A** and **2A–C.**

In comparison to controls, application of extract from female glands affected both the proportion of plants showing HR (HR vs. no HR) and the proportion of those plants showing medium or strong HR (no and weak HR vs. medium and strong HR) (X_2_^2^ = 15.9, *p <* 0.001, X_2_^2^ = 23.7, *p <* 0.001, respectively, **Figure 3A**). As shown by pairwise comparisons, in both cases HR occurrence or intensity was significantly higher when plants were treated with secretion of mated females (*p* < 0.016, Bonferroni corrected, **Figure 3A**). The application of BC on the plants also affected HR and its intensity (X_2_^2^ = 15.9, *p <* 0.001, X_2_^2^ = 23.7, *p <* 0.001, respectively, **Figure 3B**). In comparison to control plants treated with ethanol only, occurrence of HR was significantly higher at low concentration of BC (0.5 ng/100 μl ethanol; *p* < 0.008, Bonferroni corrected), but not when the concentration of the chemical was higher (*p* > 0.05, **Figure 3B**). The probability of medium or strong HR was significantly higher when plants were treated with the chemical in comparison to controls (*p* < 0.008, Bonferroni corrected), but differences among plants treated with different concentrations were not significant (*p* > 0.3).

### Location of Elicitor of OIPVs and Effect of Mating and Male-derived Compounds

**FIGURE 4:**
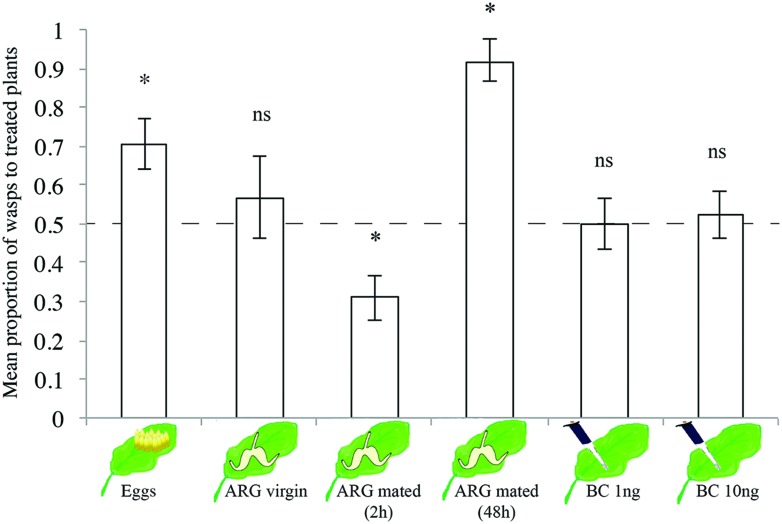
**Proportion (mean ± SE) of female *Trichogramma evanescens* wasps choosing volatiles of differently treated *B. nigra* plants when tested against untreated or solvent-treated control plants in a Y-tube olfactometer**. Each treatment, (1) *P. brassicae* eggs 24 h after egg deposition, (2) ARG (of virgin *P. brassicae* females, (3) ARG of mated *P. brassicae* females 2 h after mating, (4) ARG of mated *P. brassicae* females 48 h after mating, (5–6) BC in two concentrations (1 and 10 ng/100 μl ethanol) was replicated with four to seven plant pairs and 20 wasps per plant pair (*n* = 80–140 wasps per treatment). ^∗^*p* < 0.05, ns, not significant (*p* > 0.05); one-sample *t*-test. The dashed line indicates 0.5 = no preference. For statistical analysis, data were first arcsine transformed and then back-transformed for presentation.

We tested volatiles of differently treated *B. nigra* plants against volatiles of control plants and their effect on the attraction of *T. evanescens* wasps. The wasps showed a preference to volatiles of plants treated with either *P. brassicae* eggs (One-sample *t*-test, *t*_9_ = 3.10, *p* = 0.01, **Figure 4**) or ARG secretion of mated *P. brassicae* females (*t*_7_ = 4.94, *p* = 0.002, **Figure 4**) when tested against clean or PBS-treated control plants, respectively. Volatiles of plants treated with ARG secretion of virgin females did not attract the wasps (*t*_11_ = 0.66, *p* = 0.52, **Figure 4**). Volatiles of plants treated with secretion of females that mated in the previous few hours before testing (they would need few days to be able to start laying eggs) were significantly less attractive than the control plants (*t*_9_ = 3.05, *p* = 0.01, **Figure 4**). Neither application of 1 ng of BC (*t*_11_ = 0.07, *p* = 0.95, **Figure 4**) nor 10 ng of BC (*t*_11_ = 0.44, *p* = 0.67, **Figure 4**) induced *T. evanescens* attracting volatiles when tested against ethanol-treated control plants.

### Induction of Defense Genes Related to JA and SA Pathways

**FIGURE 5:**
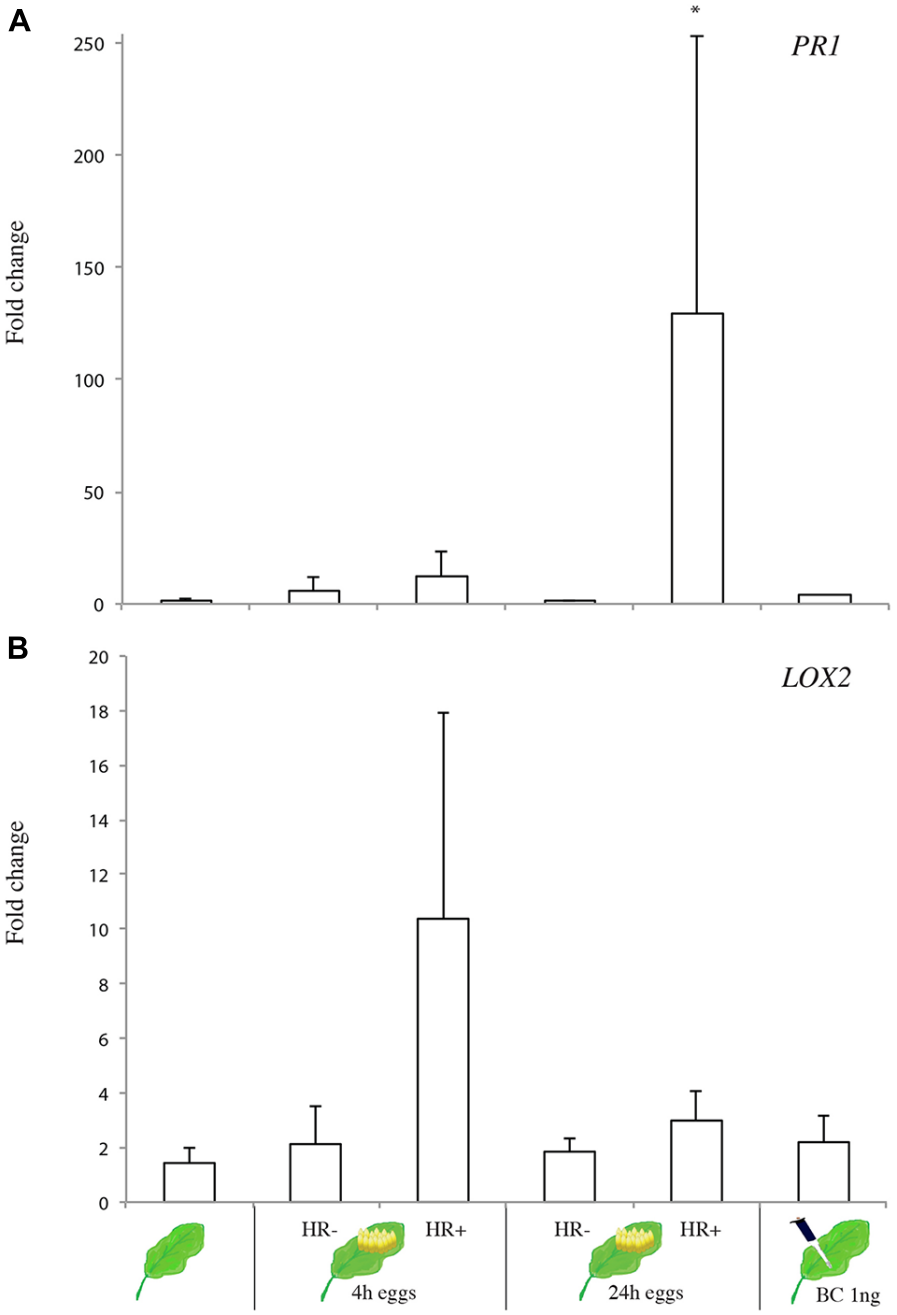
**Gene expression of **(A)***PR1* and **(B)***LOX2* genes in *B. nigra* plants exhibiting a HR-like necrosis (HR+) or not (HR-) infested by *P. brassicae* eggs (4 and 24 h after oviposition) or in plants treated with BC (1 ng)**. Bars represent mean expression levels normalized (relative to the reference gene *GAPDH*) as 2^-ΔΔCt^ with standard error bars (*n* = 3–4 biological replicates). Asterisk indicates significant difference (ANOVA *P* < 0.05; Dunnett *post hoc* test).

We measured the expression of two marker genes in the JA pathway (*LOX2*) or SA pathway (*PR1*) shown in previous studies on *A. thaliana* (Bell et al., 1995; Jirage et al., 2001). Deposition of *P. brassicae* eggs induced stronger expression of *PR1* at 24 h after oviposition in plants that expressed HR (*F*_5,25_ = 2.81; *p* = 0.044; **Figure 5A**), and this effect was neither observed in non-HR plants nor at an earlier time point. Expression levels of *LOX2* were not affected by *Pieris* oviposition, although there was a trend of *LOX2* induction in HR-expressing plants 4 h after oviposition (*F*_5,25_ = 1.47; *p* = 0.242; **Figure 5B**). Application of the anti-aphrodisiac BC did not induce expression of these two genes.

## Discussion

In the present study we show that secretion of ARGs of *P. brassicae* butterflies elicits both a direct (HR-like necrosis) and an indirect (emission of parasitoid-attracting OIPVs) defense response in the wild crucifer *B. nigra.* More importantly, we have identified one compound that plays a role as an elicitor. These results are in line with our previous studies that showed that ARG secretions of *Pieris* butterflies induce another type of defense, namely substrate-born chemical plant cues retaining *Trichogramma* wasps on egg-laden Brussels sprouts leaves (Fatouros et al., 2008, 2009; Pashalidou et al., 2010).

Exocrine secretions associated with egg deposition have been found to trigger plant defenses. For example, exocrine secretions either covering the eggs or used to glue eggs on the plant surface obtained from different insect species have been shown to elicit plant responses that resulted in egg mortality or in the emission of parasitoid-attracting plant cues (Hilker and Fatouros, 2015 and **Figure 3** therein). Although the specific elicitors were not determined, leaves of *Zea mays* exhibit indirect defenses in response to eggs of different stemborer moths including *Sesamia nonagrioidis* and *Chilo partellus.* In the former, responses were found when both the ARG secretion and the eggs dissected from the ovaries were applied, whereas in the latter responses were triggered only after application of egg-associated secretions (Tamiru et al., 2011; Salerno et al., 2013). Similarly, dissected reproductive tracts of the smaller tea tortrix *Adoxophyes honmai* comprising of ovaries, oviduct, and accessory glands (see also **Figure 1**), were shown to elicit substrate-born chemical plant cues that retained the egg-larval parasitoid *Ascogaster reticulata* on tea leaves. Unlike in the case of the stemborer moth, *A. reticulate* crushed eggs did not elicit an indirect plant response (Deshpande and Kainoh, 2012).

So far, only few studies on oviposition-induced plant defenses have identified the herbivore-associated elicitor. Bruchins isolated from pea weevils (Doss et al., 2000) and phospholipids isolated from rice planthoppers (Yang et al., 2013) have been shown to induce direct egg-killing plant defenses. Our previous studies demonstrated a role of the anti-aphrodisiac compounds BC and indole (in the butterflies *P. brassicae* and *P. rapae*, respectively) in eliciting substrate-borne chemical plant cues retaining *Trichogramma* wasps onto egg-infested *B. oleracea* leaves. Both compounds were detected in minute amounts in the egg-associated secretion of mated *P. brassicae* females whereas they were absent in secretion of virgin females (Fatouros et al., 2008, 2009; Pashalidou et al., 2010). Unlike *B. oleracea*, where wasps are retained on the leaf of an egg-infested plant, *B. nigra* plants emit volatiles that attract the wasps from a distance and provide a long-range cue to locate the host. These differences in parasitoid-attracting cues could be due to, e.g., differences in the morphology of the two plants. *B. oleracea* has a glabrous waxy leaf surface and local changes in the plant might be a strategy to improve efficiency of foraging parasitoids. *B. nigra*, on the other hand, has non-waxy leaves covered with trichomes. Plants use trichomes as direct defenses against herbivores which are also known for their negative effects on predators and parasitoids (Wei et al., 2013; Riddick and Simmons, 2014). Moreover, the cultivar *B. oleracea* var. *gemmifera* could have lost the OIPV trait due to a trade-off when breeding the species for other traits, e.g., yield or taste (Palmgren et al., 2015). For example, herbivore-induced volatile emission was found to be very rare in commercial hybrids of maize but common in farmer-selected landraces (Tamiru et al., 2011, 2012, 2015).

In the present study, we found that the anti-aphrodisiac compound of *P. brassicae* plays a role in eliciting a direct defense trait in the form of HR-like necrosis, whereas indirect defense was not triggered. Thus, our study suggests that male-derived compounds other than the anti-aphrodisiac itself may also act as elicitors. In many butterflies, males transfer a so-called nuptial gift to the female’s bursa copulatrix during mating. The male ejaculate consists of a spermatophore containing sperm, accessory gland products and a diverse array of nutrients that are also associated with egg production (Wiklund et al., 1993; Karlsson, 1998; Gillott, 2003). In some lepidopteran species, chemicals of paternal origin are transmitted to the mothers by seminal infusions that act as egg defenses against natural enemies (Eisner et al., 2002). For example, pyrrolizidine alkaloids (PAs), secondary metabolites from host plants are transmitted by males of arctiid moths to the females and they partly supply the paternal PAs to the eggs in order to deter predators (Dussourd et al., 1988; Eisner et al., 2002). Eggs *of P. brassicae* were shown to contain mustard oils, namely allylisothiocyanate (Aplin et al., 1975), but whether they are transmitted to the females during mating has not been studied yet. In our work, ARG secretions of recently mated females induced the emission of volatiles that repelled the *Trichogramma* wasps. Since the butterflies commonly start laying eggs 2 days after mating (David and Gardiner, 1962), this suggests that a physiological ‘switch’ initiated by the mating event triggers changes in the composition of the gland secretions through time. Seminal fluid proteins (SFPs) produced in reproductive tract tissues of male insects and transferred to females during mating induce numerous physiological and behavioral post-mating changes in females (Gillott, 2003; Avila et al., 2011). Thus, we cannot exclude that those physiological changes stimulated by seminal fluids are causing the observed changes in eliciting function of the egg-associated secretions after mating.

Current knowledge on egg-induced plant responses indicates a role of two antagonistic pathways, the JA and SA pathways (Reymond, 2013). In *Arabidopsis*, eggs of *P. brassicae*, and *P. rapae* consistently induce the SA signaling pathway including different *PR* genes although a necrosis is not visible in the Col-0 ecotype (Little et al., 2007; Bruessow et al., 2010). We show here that the HR-like necrosis is strongly associated with the induction of the SA-responsive gene *PR1*, which confirms our earlier observations on *P. rapae*-egg induced HR in *B. nigra* (Fatouros et al., 2014). *LOX2*, a gene involved in the JA-signaling pathway in *Arabidopsis*, was not affected by egg deposition in *B. nigra* and only slightly but not significantly induced at 4 h after oviposition by *P. brassicae* when HR-like necrosis was present. Yet, egg deposition by the conspecific *P. rapae* induced *LOX3* at 24 h after oviposition on *B. nigra* plants in both phenotypes (HR+/HR-) (Fatouros, unpublished data). This suggests that the JA-dependent wound-induced response might work in parallel to an SA-dependent egg-induced response (Reymond, 2013). The current study supports this hypothesis and adds an extra aspect to explain the variability of the observed response in *B. nigra*: the presence or absence of an HR-like necrosis.

## Conclusion

Our study demonstrates that secretions obtained from ARG reservoirs of *Pieris* butterflies contain specific elicitors of the different plant defense responses against eggs in *Brassica*. Mating changed the eliciting effect of the secretion suggesting that either male-derived compounds that arrive in the secretions or physiological changes triggered by mating play a role. Anti-aphrodisiac compounds of *Pieris* butterflies transferred during mating that were shown to induce substrate-borne chemical cues in *B. oleracea* plants did only partly induce egg-killing defenses in *B. nigra*. As a direct egg-killing trait, HR-like necrosis, involves expression of *PR1* defense genes and most likely the SA-dependent pathway. Other studies on OIPVs have shown that the JA-pathway is involved (Meiners and Hilker, 2000; Hilker et al., 2002; Mumm et al., 2003). It is rather unlikely that the same compounds in an egg-associated secretion elicit different defense pathways and responses. Our study adds to the information on HAMPs and increases our understanding of how plants recognize information from herbivores in a non-feeding stage to respond with insect-killing defenses before actual damage occurs.

## Conflict of Interest Statement

The authors declare that the research was conducted in the absence of any commercial or financial relationships that could be construed as a potential conflict of interest.
